# Supplementary material for: Are carbon emissions trading and green financial instruments synergistic? -Comprehensive quantitative research based on content analysis
Source: PLoS One. 2024 Mar 7;19(3):e0298601. doi: 10.1371/journal.pone.0298601 (PMC10919601; doi:10.1371/journal.pone.0298601)
Supplement: S1 Data — (ZIP) [file pone.0298601.s001.zip › Data/地级市Z_F工作报告.zip!/╣π╬≈/╟╒╓▌╩╨/└┤╫╘╣1⁄2╓┌║┼í╛┬φ┐╦╩2╛▌═°í┐_2016.docx]

各位代表：

现在，我代表市人民政府向大会报告工作，请各位代表审议，并请市政协委员和列席会议的同志提出意见。

“十二五”时期经济社会发展回顾

“十二五”时期，发展形势一年比一年严峻。这五年，是我市负重前行、爬坡过坎艰难的五年，是我市克难攻坚、行稳致远奋斗的五年，更是我市抢抓机遇、跨越发展收获的五年。面对国际金融危机持续影响、“三期叠加”等一系列重大风险挑战，面对多次自然灾害的袭击，在市委的坚强领导下，全市各族人民积极适应经济发展新常态，凝心聚力、沉着应对，深入实施“建大港、兴产业、造新城、强科教、惠民生”发展方略，基本完成“十二五”目标任务，综合实力、战略地位、产业发展、城市建设、民生保障等方面实现历史性嬗变。回首五年，办成了一批大事喜事。

——人均gdp首次跨上5000美元台阶，财政收入突破160亿元，一般公共预算收入突破50亿元，外贸进出口总额突破50亿美元，经济发展迈入新阶段。

——中石油一期1000万吨炼油等重大项目全面投产，规上工业总产值突破1000亿元，钦州港经济技术开发区成为百亿元税收园区，产业发展进入重大收获期。

——港口吞吐能力突破1亿吨，集装箱吞吐量近100万标箱，连续5年居北部湾沿海港口首位，建成运营3条高速铁路，实现亿吨大港梦想，迈进了高铁时代。

——中马产业园开创“两国双园”国际合作新模式，保税港区全面封关运营，以钦州为核心的北部湾自由贸易试验区启动申报建设，成为国家级平台及享受政策优惠最多的地级市之一。

——启动滨海新城建设，拉开现代滨海城市框架，城市功能进一步完善，成功创建国家园林城市，城市品位大幅提升。

——县域工业驶入快车道，县域规上工业总产值突破800亿元，与临港工业形成并驾齐驱的发展新格局。

——北部湾大学一期建成启用，建成广西北部湾地区最大职教园区，教育发展迈出新步伐。

——成功举办广西第十二届运动会和广西第十三届少数民族传统体育运动会，极大提升知名度和影响力。

五年来，顺应人民过上美好幸福生活的新期待，我们风雨兼程、迎难而上，我们锐意进取、砥砺奋进，一步一个脚印，一年一个台阶，迈出了坚实步伐，谱写了崭新的篇章。

一、这是全市经济快速攀升，综合实力实现新跨越的五年

坚持把扩量提质作为加快发展的主要任务，不断壮大经济规模，gdp、财政收入、固定资产投资、港口吞吐量、社会消费品零售总额等翻一番，工业产值、外贸进出口总额、集装箱吞吐量等翻两番。gdp年均增长11.5%，财政收入年均增长22.7%，规上工业总产值年均增长30.2%，固定资产投资累计完成3413亿元，外贸进出口总额年均增长34.7%。

二、这是亿吨大港和多个国家级平台建成，战略地位实现新飞跃的五年

（一）集装箱枢纽港已现雏形。深入实施亿吨大港工程，全面启动大榄坪50平方公里整体开发，建成30万吨级主航道及码头，五年新建成码头泊位35个，吞吐能力达到1.14亿吨。开通运营至昆明集装箱海铁联运“五定”班列，新开通16条班轮航线。五年累计完成吞吐量2.93亿吨，年均增长16.6%，集装箱完成312.1万标箱，连续5年稳居北部湾各港首位，年均增长30.3%。

（二）开放平台取得新突破。成功搭建了一批国家级平台。中马产业园获国务院批准设立并获得国务院专门政策支持，启动区基础设施和配套体系基本形成。保税港区全面封关运营，整车、粮食、肉类进口口岸获批并开放启用，全国首家进口酒类知名品牌示范区获批。中国—东盟港口城市合作网络全面启动，中国—东盟港口物流信息中心一期建成。率先推进以钦州为核心的北部湾自由贸易试验区申报，成功复制推广了多项自贸区政策。

（三）集疏运体系优化升级。完善出海通边、服务中南西南的大通道建设，建成运营钦州至南宁、至北海、至防城港3条高速铁路、4条普通铁路和钦州港至六景、钦州至崇左2条高速公路，黎钦铁路扩能改造、钦州港至大榄坪、大榄坪至保税港区铁路通车，钦州支线机场启动选址研究，广西沿海交通主枢纽地位进一步凸显。

三、这是临港与县域产业双轮驱动，产业结构实现新升级的五年

（一）临港产业快速崛起。深入实施千百亿产业“崛起工程”和“攻坚工程”。建成投产中石油一期，成功引进煤基多联产等项目，石化产能超过1000亿元。中船修船资源整合、力顺轻型载货汽车开工建设，装备制造业加快培育。中粮油脂等项目投产，国投二期、热电联产等项目加快建设，粮油食品、木材加工业产值双双超100亿元，造纸及纸制品、能源及新能源、新材料等百亿元产业加快形成。5年新增亿元企业148家，比2010年增长3.3倍。

（二）县域工业突破壮大。先后实施县域工业“突破工程”和“壮大工程”，县域工业成为经济增长主力军。天山微电子、裕源药业、明利化工、邦琪药业等龙头企业壮大发展，医药、制糖、饲料、编织、纺织、服装、建材等县域特色产业集群加快形成。县域规上工业总产值达到886亿元，5年增长5倍，规上工业企业达到290家。

（三）高新产业加快培育。建成自治区级高新区，卓能新能源、富仕通等重点项目建成投产，慧宝源基本建成，电子信息和医药产业初具规模。建成自治区级科技企业孵化器，5年新增9家自治区级企业技术中心、35家高新技术企业。发明专利拥有量5年增长10.5倍，高新技术企业工业总产值由2010年的753万元增长至62亿元，几乎从空白起步，5年增长800多倍。

（四）服务业迅速壮大。引进一批知名零售巨头，建成一批大型商贸市场，电子商务方兴未艾。“引银入钦”成效明显，银行业机构入驻数居全区前列。与全国中小企业股份转让系统签订战略协议，润港林业成功在新三板上市。在全区率先开展跨境人民币贷款业务，跨境人民币结算量达38.2亿元，中马产业园获批试点资本金意愿结汇。旅游人数突破1000万人次，旅游收入突破100亿元。加工贸易异军突起，占全区加工贸易总额三分之一。

（五）特色农业提质升级。深入实施“特色农业提升工程”， 形成7大养殖基地，奶水牛、大蚝等养殖规模全国第一，10个特色农业产品获得国家地理标志产品保护。粮食生产实现“五连增”。率先完成集体林权制度主体改革，林下经济走在全区前列。5年新增农民合作社578家、重点龙头企业42家，建成国家农业综合开发现代农业园1个、国家农业科技园区1个、广西现代特色农业（核心）示范区1个。

四、这是滨海新城开发建设全面启动，城乡面貌实现新变化的五年

（一）城市发展拉开框架。滨海新城崭露新形象，白石湖、沙井岛、茶山江、辣椒槌四大功能片区基础设施初步完善，建成一批重大功能项目。河东新城建成体育中心及配套设施，一批上规模上档次的商住小区展现新风貌。建成白石湖公园、林湖公园、中山公园、江滨公园等一批市民休闲场所。建成子材大桥、北部湾大道、扬帆大道，“一环二横四纵一联”的城市快速路网络加快建设。旧城区功能提升，形成一批新商圈。新城开发、旧城改造和县镇建设同步推进，灵山江北新区、浦北金浦新区、钦北新城加快建设。

（二）“四城联创”成效显著。实施“园林生活十年计划”，先后成功创建自治区和国家园林城市。打通166条城区“断头路”，建成一批小街小巷惠民工程。统筹推进城市环境综合整治，成功创建广西卫生城市、文明城市，获得全国文明城市提名，全面开展广西食品安全城市创建工作。市政设施不断完善，建成城区应急水源，实施城区污水管网清淤疏浚工程，完成41个排水改造工程，城市防洪排涝治理初显成效。获批全国第三批智慧城市试点。

（三）生态文明水平提高。推进节能减排重点工程建设，淘汰落后产能171.4万吨，完成主要污染物排放总量控制目标。重金属污染防治通过验收。石化产业园获批全国首批国家级园区循环化改造示范试点。建成河东、大榄坪和10个重点镇污水处理厂，城市生活污水处理率94.8%。加强茅尾海综合治理，主要河流水质达标率、饮用水源地水质达标率均达到100%，空气质量优良率提升至全区第三位。森林覆盖率53%。持续推进“美丽钦州”乡村建设活动。

五、这是民生改善力度持续增强，群众福祉实现新提高的五年

（一）基本公共服务全面加强。民生支出逐年增加，连续5年占财政支出比重80%以上。坚持教育优先发展，建成一批中小学、幼儿园，钦州学院获自治区批准筹建北部湾大学并实现整体搬迁。公办基层医疗卫生机构全部实施基本药物制度，新增2家“三甲”医院。建成市老年大学新校区、244个村级公共服务中心、979家农家书屋，公共文化场馆全部免费开放。坭兴陶烧制技艺和跳岭头列入国家非物质文化遗产，“扫黄打非”进基层经验全国推广。完成282座病险水库除险加固，建成沼气池5万座，解决了123万群众的饮水安全问题，完成县镇村道路通畅三年大会战任务，100%行政村、50%以上自然村通水泥路。创新社会治理方式，推进社会治安防控信息化建设和网格化管理，社会治安大局稳定，公众安全感稳步提升。基础设施、公共服务、社会管理进一步向农村延伸。

（二）社会保障体系不断完善。在全区率先实现社保大集中信息管理，率先试点城乡居民大病保险，实现全区定点医疗机构异地就医即时结算，三项医疗保险参保率95.1%。健全失地失海农渔民养老保障机制，城乡低保月人均补助水平分别提高到282元和142元，实现动态应保尽保。人口增长得到有效控制，出生人口素质进一步提高。城镇新增就业12万人，比“十一五”增长368%，城镇登记失业率2.68%。建成保障性住房26206套，农村危房改造43560户。城镇居民人均可支配收入、农民人均纯收入分别年均增长9.5%、13.4%。

（三）扶贫攻坚取得阶段性成效。结对帮扶119个贫困村，在全区率先创建扶贫产业示范园，构建了“三位一体”大扶贫格局。5年共投入扶贫财政资金6.4亿元，修建贫困村道路1178条，实现了所有贫困村通硬化路，解决了107.8万村民行路难问题。5年减少贫困人口20.5万人。

各位代表，刚刚过去的2015年，是2008年以来宏观经济形势最为严峻的一年，面对前所未有的经济下行压力和严峻复杂的困难挑战，全市上下齐心协力、奋力作为，深入贯彻落实中央和自治区稳增长决策部署，及时出台稳增长46条措施，推动经济稳中向好。全年gdp增长8.4%，规上工业总产值增长5.9%，财政收入增长17.3%，固定资产投资增长22.9%，外贸进出口增长9.2%，集装箱吞吐量增长34%。gdp、固定资产投资增速居全区前列，财政收入增速全区第一，较好完成了市四届人大六次会议确定的目标和任务，“十二五”规划实现圆满收官，广大人民群众有了更多获得感。

一年来，我们重点抓了几项工作。

——抓重大项目建设。深入实施“项目推进和招商引资攻坚年”活动，实行市领导联系重大项目制度。加大投融资创新力度，组建白海豚投资基金，组织开展4批国家专项建设基金项目储备和申报，落实北部湾重大产业发展专项资金，出台了ppp实施意见，首次成功发行中期票据、非公开定向债务融资工具等金融产品。盘活调整用地1537公顷。围绕重点产业开展精准招商，全年新签亿元以上项目132个，引进内外资506亿元；总投资500万元以上新开工项目增长52.1%，竣工项目增长30.4%。

——抓精准服务企业。深入实施“企业服务年”活动，坚持“市长企业接待日”活动，强化“一企一策”精准帮扶，实施对铁合金用电大户给予每度电补贴2分钱等一揽子政策，争取到获批直供电试点，发放“惠企贷”1.6亿元，工业经济加快回升，新增规上工业企业30家、亿元企业23家。出台新购商品住房契税补贴等刺激政策，全面实行棚户区改造“以购代建”和公租房“以租代建”，着力去库存，房地产消费稳中有升，建筑业壮大发展。

——抓城市功能提升。深入实施“城市建设年”活动，城市配套功能加快完善，解决了扬帆大道、蓬莱大道等“断头路”、“半边路”征拆“老大难”问题。上沙、沙坪等城中村改造稳步推进。完成100个生活污水直排口截流截污工程。

——抓改革创新驱动。开展“改革创新年”活动，全面推进商事制度改革，深化行政审批制度改革，大力推进行政管理体制改革。中马产业园获批在全区率先试点设立外商股权投资类企业。保税港区完成委托管理，产业金融等特色业务取得突破发展，通用航空项目加速推进，整车进口1756辆。发布市直部门第一批权力清单和责任清单，全面完成市级行政审批事项清理。

——抓社会民生改善。投入43亿元全面完成为民办十件实事工程。编制完成主城区教育布点规划，制定解决主城区“入学难”三年攻坚六年行动计划，新建、迁建学校30所，人和小学、市职教中心幼儿园等一批学校建成使用，市三中成功创建自治区示范性高中。全年城区环境空气质量优良率达90%以上。“三农”工作成效明显，完成2.1万亩“双高”糖料蔗基地建设，成为广西首个速丰林采伐管理改革试点市和水产养殖保险试点市。精准脱贫识别工作扎实推进。

五年来，我们认真执行市人大及其常委会的决议和决定，自觉接受人大、政协和社会监督，认真办理人大代表建议和政协提案。严格贯彻中央八项规定，深入开展党的群众路线教育实践活动和“三严三实”专题教育，全面推进依法行政，政府自身建设全面提升，行政机关和干部作风明显好转，政府执行力和公信力不断提高，政务环境持续改善。国防教育和后备力量建设深入开展，双拥共建工作取得新成效。数倍获得新增建设用地计划指标，国土资源、就业、知识产权保护、防震减灾、档案、保密等工作荣获全国先进，海关、检验检疫、海事、边检、消防、税务、工商、质监、供电、烟草、通信、邮政、盐务、气象、水文等为我市经济社会发展作出了新的贡献，民族、宗教、侨务、统计、监察、审计、国资监管、物价、农机、供销、人防、海防、口岸、水库移民、科普、政务督查、哲学社会科学、决策咨询、地方志、法律援助、残疾人和关心下一代等工作取得新成绩，工会、共青团、妇联等群团组织在经济社会发展中发挥了重要作用。

各位代表，“十二五”发展成绩令人振奋，经验弥足珍贵。我们深深体会到：必须坚持党的领导，不断深化市情认识；必须坚持发展为要，在发展中转变调整；必须坚持改革开放，用以解决矛盾问题；必须坚持统筹兼顾，注重协调互动发展；必须坚持民生为本，让成果最大程度惠及于民。

各位代表，“十二五”取得的成绩来之不易，得益于自治区党委、自治区政府和市委的正确领导，得益于历届党委、人大、政府、政协和老同志打下的坚实基础，得益于市人大的有力监督和市政协的大力支持，得益于市内外朋友的鼎力帮助，得益于全市各族人民的团结奋斗、顽强拼搏。在此，我代表市人民政府，向为钦州发展付出辛勤劳动的全市各族人民、人大代表、政协委员，向各民主党派、工商联、各人民团体和社会各界人士，向自治区各部门、中直区直驻钦单位、驻钦部队、武警官兵和兄弟城市，向所有关心支持钦州发展的港澳台同胞、海外侨胞和海内外朋友，表示崇高的敬意和衷心的感谢！

在肯定成绩的同时，我们也清醒地认识到，“十二五”时期，我市经济社会虽然实现较快发展，但当前经济社会发展中还存在不少困难和问题。主要是：开放平台未能充分发挥作用，辐射带动效应有限；经济总量偏小、发展基础相对薄弱，人均gdp低于全国、全区平均水平，与全面建成小康社会目标差距较大；农业比重偏大，工业化率较低，产业结构较单一；疏港体系尚未完善，港口物流成本较高，大通道效应不明显；基本公共服务水平有待提高，政府自身建设有待加强等。下一步，我们将围绕落实国际大通道、战略支点和重要门户“三大定位”，按照中央、自治区的决策部署，坚守自身特色，坚持战略提升，突出开放合作，不断构建继承式创新发展的战略体系，全面提升城市综合竞争力和战略地位，努力把各项工作做得更好，不辜负人民群众对更加美好生活的期盼！

“十三五”时期发展思路和主要目标

各位代表，未来五年是钦州建设现代生态滨海城市、实现发展新跨越的攻坚时期，是全面建成小康社会的决胜阶段。

“十三五”时期的指导思想是：高举中国特色社会主义伟大旗帜，以马克思列宁主义、毛泽东思想、邓小平理论、“三个代表”重要思想、科学发展观为指导，深入贯彻落实习近平总书记系列重要讲话精神，全面贯彻党的十八大和十八届三中、四中、五中全会精神，落实“四个全面”战略布局，坚持创新、协调、绿色、开放、共享的发展理念，紧紧围绕中央赋予广西的三大定位和自治区的战略部署，大力实施“开放创新、港城联动、产业强市、生态惠民”发展战略，建通道、扩总量、提质量，实现跨越式发展，基本建成“一带一路”有机衔接的重要门户港、区域性国际合作新高地，基本实现“十年巨变”目标，全面建成小康社会。

“十三五”时期的主要目标任务是：在全区开放发展格局中提档进位、奋力争先，实现与全国同步全面建成小康社会目标。

——经济总量质量效益再上新台阶。2020年，地区生产总值力争突破2000亿元，比2010年翻两番，人均生产总值力争突破5.7万元，财政收入突破300亿元，一般公共预算收入突破100亿元，规上工业总产值力争突破4000亿元，固定资产投资5年累计完成6500亿元。经济总量在全区进位靠前，经济效益同步提升，经济结构优化升级取得更大进展。

——“一带一路”有机衔接重要门户港地位确立。争取获批设立北部湾自由贸易试验区并建成核心区，建成区域性国际航运中心和北部湾集装箱干线港，连接东盟与西南中南地区的通道枢纽作用进一步增强，港口物流、集装箱业务加速发展，现代航运服务体系基本建立。2020年，港口吞吐量力争达到1.5亿吨，集装箱吞吐量力争达到500万标箱。

——区域性国际合作新高地基本形成。建成中国—东盟港口城市合作网络钦州基地，中马产业园、保税港区等开放平台合作体制机制进一步完善，平台作用充分发挥，成为中国—东盟自贸区升级版先行试验区，形成全方位开放型经济发展格局。

——现代产业体系更加优化。产业布局更趋合理，大型临港产业支柱作用进一步凸显，以港口物流为核心的现代服务业加快集聚发展，建成一批具有全国或区域性影响的重要产业基地，成为北部湾沿海现代产业集聚核心区。

——现代滨海城市形象全面彰显。港城、产城、学城融合进程加快，建成国家生态园林城市和智慧城市，北部湾体育运动休闲基地特色凸显，新型城镇化水平和质量显著提高，全市常住人口城镇化率达到45%，努力把钦州建设成为生机勃发、活力涌动的中国最美内海城市。

——人民生活水平和质量显著提高。2020年，城乡居民收入比2010年翻两番，比2015年翻一番，高于全国、全区平均水平。基本公共服务均等化水平显著提升，城乡社会保障体系更加完善，就业更加充分，全面完成精准脱贫攻坚任务，发展成果更多惠及百姓。

——体制机制更有活力。全面深化改革、法治建设深入推进，重点领域和关键环节改革取得重大成果，法治政府基本建成，创新驱动能力增强，市场体系更加健全，社会充满活力创造力。

——生态钦州品牌打响。生态型经济体系形成规模，生态保护和环境治理加强，区域生态环境质量保持良好，生态保护屏障基本建成，绿色低碳循环生产生活方式加快普及，节能减排约束性目标任务全面完成，建成国家森林城市，生态钦州建设成果显著。

各位代表，“十三五”发展的宏伟蓝图已经绘就，尽管前进道路上面临着许多困难和挑战，只要有自治区党委、自治区政府和市委的正确领导，有市人大、市政协的监督支持，有全市广大干部群众的共同努力，规划所确定的目标就一定能全面实现，一定能够为全市人民创造更多的福祉！

2016年主要目标与重点工作

2016年是“十三五”规划的开局之年，开好局、起好步，对“两个建成”宏伟目标的实现至关重要。

经济社会发展的主要预期目标是：地区生产总值增长10%；财政收入增长6.5%；固定资产投资增长16%；规上工业总产值增长9.5%；外贸进出口总额增长10%；港口吞吐量6800万吨，其中集装箱130万标箱；pm10、pm2.5分别下降5%以上。

围绕上述目标，我们将突出“特色+绿色”发展，充分发挥平台、区位、后发优势，着力破解资金、交通、人才、体制机制瓶颈，抓好供给侧结构性改革，加快发展海洋经济、平台经济、县域经济、知识经济、生态经济，大力实施创新驱动、财税金融、产业拓展、精准脱贫、城市经营、营销招商、土地运营、智慧钦州、环境提升、法治建设等10个重大行动，重点抓好以下几个方面的工作：

一、坚持开放引领，提升发展格局

（一）大力发展平台经济

全面推进中马产业园开发建设。进一步完善“两国双园”架构，推进基础设施、产业引进和体制创新。重点建设马莱大道、友谊大道北段等项目，完善园区商业、医疗、教育等公共服务功能。扩大高端产业集聚，推动慧宝源投产，开工建设中马科技园、智慧产业园、易通浩光电产业园、保利协鑫分布式能源等项目，争取超高压输变电装备制造、3d打印、稀土铝合金电缆等项目落户。规划建设跨境服务业集聚区，推进跨境金融服务中心建设，建立完善以资本为导向的园区开发模式。完善园区治理体系，探索建立法定机构管理体制和模式，加快建设自治区改革创新先行园区。

拓展保税港区功能。继续完善配套基础设施，建设九大街、大型集装箱检查设备等项目。扩大整车进口、进口酒类展示等特色业务，推进开展跨境电子商务进口业务、粮油交易业务，推动水果快线项目落地，发展汽车展示销售市场，引进一批外贸综合服务企业，培育粮食、石材、木材等大宗商品进口交易，争取平行进口汽车试点。大力培育通航、冷链物流产业，建设通用航空产业基地，争取进境水果、活牛进口等指定口岸获批。探索实行码头资源共享与同港同船作业，争取国际船舶登记、启运港退税等政策试点。实施加工贸易倍增计划，加强与东盟国家贸易往来，促进外贸繁荣发展。

提升发展钦州港经济技术开发区。推动“四区联动”发展，突出专注经济发展的功能定位，统筹解决好历史遗留问题和加快发展的关系。开展国家级经济技术开发区扩区研究，加快产业项目和基础设施建设，抓好千万吨级炼化一体化、芳烃、煤基多联产、国投三期等项目前期工作，加快行政商务中心、综合物流加工区建设。大力开展国际港航服务和产业合作，推进建设北部湾现代港航物流中心。

加快建设中国—东盟港口城市合作网络钦州基地。制订发布合作网络章程和五年行动计划。争取成立合作网络秘书处，将合作网络纳入中国—东盟合作机制协调范畴。力争与已开通航线的港口城市全部实现港航信息互联。建成第一批项目，策划实施第二批项目。扩大与东盟港口城市的友好交流合作。

争取获批北部湾自由贸易试验区。做好钦州片区土地总规、城市总规调整审核等工作，启动自贸区建设规划研究，配合自治区争取尽快获批。围绕复制推广的政策，继续开展先行先试。

（二）全面推进大通道建设

加快大能力码头泊位建设。围绕打造北部湾集装箱干线港，深化与新加坡国际港务等国际港航企业合作，全面加快大榄坪50平方公里整体开发运营。全面建成运营30万吨级油码头及航道、金鼓江航道等项目，完成东西航道清淤工程，开工建设鹰岭作业区2#泊位等工程，加快推进大榄坪9—13#泊位等一批重大码头项目，开展10万吨级航道扩建、20万吨级集装箱航道及码头等项目前期工作，启动大环、三墩扩区二期等港口新作业区开发，拓展港口新增长点。

提升互联通达能力。开工建设保税港区集装箱办理站、东站经中马产业园至大榄坪及保税港区至三墩铁路、保税港区车场和货场，完善大榄坪港区海铁联运配套运输设施，降低集装箱运输物流成本。提升港口集箱能级，优化沿海三港穿梭巴士，加快北部湾港外贸航线转移，加密集装箱航班，继续培育开通至东盟国家的直航航线。实质性运营贵阳“无水港”，培育建立昆明、成都等“无水港”，推进区内主要城市、云贵地区的集装箱集中钦州港集散。

提升港航服务能力。加强口岸联检大楼等基础设施和口岸监管设施建设，加快进出口检验检测平台建设，完成鹰岭、勒沟作业区围网升级改造，推进与西南、中南地区重要物流节点城市及海关特殊监管区域合作，完善“一站式”服务机制，构建集电子政务、电子商务、电子物流“三位一体”的口岸公共信息服务平台，提高通关效率。加快建设钦州港综合物流加工区，推动在钦州设立北部湾航运交易所，促进航运服务要素聚集。

二、坚持产业强市，推动扩量提质

（一）大力发展海洋经济

实施产业拓展行动，优化发展空间，打造新的经济增长点。重点发展临海重化工业、海洋运输、海洋船舶、海洋渔业、滨海旅游，培育发展海洋工程、海洋生物医药、海产品深加工、海洋服务等海洋新兴产业，促进现代化海洋产业集群发展。重点服务中石油、国投、中粮、金桂等企业稳产达产，力促一批重大项目投产。协调解决企业生产原料问题，力争泓达化工等尽快投产，天恒石化、玉柴石化满负荷生产。继续服务扶持铁合金企业恢复扩大生产，推进钦州港区粮油加工产业整合，推动更多大企业开展直供电业务。开展茅尾海蓝色海湾五年行动，加快建设北部湾综合性海洋大学，建设海洋科技成果转化平台，构建更有竞争力的海洋经济体系，争取列入全国海洋经济强市示范市。

（二）着力发展县域经济

实施县域工业提升工程，支持县区创建自治区级经济技术开发区，建设陆屋临港产业园、金窝工业园、皇马工业园等临港配套产业重点承接基地，建成投产天山微电子四期、欧艺家具、华汇新材料、卓能新能源二期等项目，更大力度开展“惠企贷”工作，发展壮大食品加工、木材加工、医药、制糖、纺织、编织等特色产业，强化质量和品牌建设，促进县域工业扩量提质。建设武利、泉水、那丽、大寺等一批重点工业镇。力争县域工业产值超过1000亿元。

（三）集聚发展现代服务业

大力发展总部经济，促进服务要素集聚。加快推进长融世贸中心、东盟商贸城等大型市场、商业综合体建设，扩大城乡消费市场。升级改造人民路、钦州湾广场、年年丰等核心商业区，加大实施“农改超”，完善商贸流通网络。大力发展电子商务，扶持东盟商贸城采购批发平台，推进农村电商发展，支持快递业发展，规划建设中马跨境电商经济合作试验区。扶持培育物流龙头企业，建设物流信息基地，启动建设北部湾航运服务集聚区，重点发展船舶运输、船舶租赁等传统航运服务，加快发展航运交易、金融保险、船舶经纪、船务代理等航运服务。着力构建特色旅游产品体系，推进三娘湾文化旅游综合开发，做好海豚主题公园等前期规划工作，主动融入北部湾和东南亚旅游圈。

（四）提升发展特色农业

继续实施特色农业提升工程，稳定粮食生产，全面推进特色产业结构优化、品质升级。大力推进自治区级、市级现代特色农业示范区建设，加快尖山欢乐农庄、康熙富硒水稻、九佰垌、龙武、浦北生态观光等现代特色农业示范区发展。强力推进“双高”糖料蔗基地建设。积极发展新的农业生产经营主体，加大龙头企业、专业合作示范社培育力度，加快发展农产品加工业，加强农产品品牌、专业市场建设，支持发展农产品储藏保鲜、分等分级、产地初加工和产品营销业务，打造农业产业链。积极完善市、县、镇三级土地流转服务体系，推进现代农业产业化、规模化经营。

三、坚持项目带动，增强发展后劲

（一）开展“重大项目攻坚年”活动

继续将重大项目作为全市经济工作的重要抓手，健全重大项目统筹推进机制，完善市领导联系重点项目制度，统筹协调服务重大项目，集中力量破解项目建设瓶颈。全市统筹推进重大项目380个，年度计划投资350亿元以上。力促每季度都有重大项目开工，加快推进热电联供、通明化工、大塘至浦北高速公路、沿海高速公路改扩建等重大项目前期工作，促进国投二期、中船修船资源整合、力顺轻型载货汽车等项目尽快竣工，加快贵合高速公路、南钦二级公路改扩建、陆屋至灵山一级公路等项目建设，形成新的增长点。

（二）开展“园区基础设施会战年”活动

实施园区基础设施提升三年攻坚，组织实施重点园区基础设施项目189个，突出抓好各类园区主干路网、关键物流通道、供电、给排水、环保设施、用地平整等基础设施和公共服务设施建设，重点推进钦州港经济技术开发区、高新区重大基础设施建设，完成园区骨架路网互联互通，搭建进出园区快速通道，近期规划区域基本达到“五通一平”。加快高新区二期启动区征地搬迁。继续完善提升县域工业园区。全面开发建设北部湾华侨投资区。

（三）实施营销招商行动

实施重点区域精准招商。创新招商机制模式，健全招商激励机制，建立与中马产业园、保税港区、北港集团招商联动机制。突出重点产业招商，瞄准行业领军企业，重点围绕石化、装备制造、现代服务业及电子信息、生物医药、新材料、新能源（汽车）等行业，引进一批龙头和下游企业，力争在海洋工程装备、机器人制造、石墨烯等领域有突破。优化招商服务环境，健全领导联系协调常态化机制，完善重大招商项目进展情况定期通报机制。优化审批服务，实行全程跟踪服务，力促项目“一站式”审批。跟踪服务投产企业，及时化解落户企业生产经营遇到的问题和困难，巩固招商成效。

（四）实施土地运营行动

创新用地保障方式。推进城乡建设用地增减挂钩、低丘缓坡荒滩等未利用地综合开发利用、小微企业创业基地标准厂房建设用地、城市低效用地再开发等土地管理改革试点，落实建设占用耕地“占补平衡”和“占优补优”，做好建设用海整体报批工作。促进土地节约集约利用，完善产业项目落户与项目投资强度、产出效益挂钩制度。提高土地运营能力，协调规划、收储、供应之间的关系，推进“绩效用地”。全面清理批而不征、征而不建的用地项目，对闲置用地项目实行退出机制，建立低效产业用地流转和二次开发奖惩制度，对收回土地重新调剂使用。完善落实征地搬迁配套政策，科学编制集体土地征收搬迁计划，以拆违促拆迁。

四、坚持城乡协调，打造宜居城市

（一）实施城市经营行动

落实房地产消费鼓励政策，加快消化商品房存量，推进棚户区改造“以购代建”和公租房“以租代建”，促进房地产平稳发展。策划组织蚝情节等重大活动。开展营销钦州活动，利用传统和新兴媒体，运用市场营销理念和方法管理城市，制定城市cis系统和城市营销规划，打造城市形象品牌。启动创建国家生态园林城市、全国防震减灾安全示范城市，举办第九届广西“园博会”。实施城市亮通工程，开展主要街区亮化、绿化、美化、环境卫生整治和规范管理，提升东站区域等城市出入口和重要节点形象。

（二）推进城市设施完善升级

全面加快滨海新城开发建设，开工建设广西海洋农业职业技术学院、国际海员俱乐部等重点项目，白石湖片区基本形成骨架路网，积极推进“海绵城市”建设。完善主城区公共服务设施，加快城市公共交通建设，实施公共自行车服务系统工程，探索引进新能源汽车和城市公共汽车的社会化运营。实施中心城区道路畅通工程，每年改造升级两条道路，进一步完善城市路网，开工建设南北二级公路（桅杆坳至清水窝），推进市区断头路、小街小巷、排水系统等改造建设。推进城区第二水源前期工作。开展申报城市地下综合管廊试点的筹备工作，力争获得国家新型城镇化综合试点。加快旧城改造，推进上沙、沙坪城中村改造。推进环境综合整治三年行动计划，加大城市综合执法力度。着力破解交通瓶颈，加快中心城区连接县城、镇村的快速交通网络建设。出台户籍、社保、教育、医疗、土地等新型城镇化配套政策，畅通农村人口有序进入城镇的通道，加快人口集聚。统筹推进城乡建设，按照产城互动要求，加快灵山新城、浦北金浦新区、钦北新城开发建设，着力培育一批特色重点镇。建成县县通天然气全线接通工程。

（三）实施智慧钦州行动

做好智慧钦州顶层规划，按照统筹规划、共享协同、迭代完善、注重实效的原则，推进国家智慧城市试点工作，加快实施智慧政务、智慧港口、智慧交通、智慧园区、智慧健康、网格化综合管理等工程，加快完成云计算中心、基础数据库、城市公共信息平台等项目前期工作，推进云视频中心、市民一卡通等项目建设，实施政务服务事项网上办理，形成智慧钦州建设的实际应用成果。创新城市管理方式，推进网格化城市综合管理体系建设，建成覆盖主城区60平方公里范围内的数字城管系统，全面提升城市管理水平。

五、坚持改革创新，激发发展动力

（一）深化重点领域改革

着力破解体制机制瓶颈。进一步推进商事制度改革，探索推进全程电子化登记和电子营业执照。加大行政审批制度改革，规范和优化审批程序和审批流程，实施重大项目联合审批制度。推进行政管理体制改革，完成市、县、镇三级政府权力清单和责任清单。进一步理顺市与城区的关系。深化农业农村改革，稳步推进农村土地承包经营权确权登记颁证工作，争取扩大政策性农业保险覆盖范围。深入推进国企、教育、卫生计生、文化、供销社、华侨农林场等领域改革，组建文化传媒集团，积极引入社会资本发展教育、文化、医疗卫生事业。

（二）实施财税金融行动

着力破解经济发展资金瓶颈。研究利用沿边金融改革政策，扩大资本项目意愿结汇、跨境人民币业务，探索设立跨境金融机构。加大财源培植力度，有效防范政府性债务风险。整合统筹使用财政专项资金，减少资金沉淀。加强投融资平台建设，盘活存量优质资产，推动融资平台转型，适当进入竞争性行业。尽快设立运营白海豚投资基金。加大企业债券和专项债券资源培育开发和储备工作，合理利用中期票据、融资租赁、非公开定向债务融资工具，组织实施一批ppp试点项目，探索产业引导基金、创业投资基金、股权融资、城市发展基金等融资方式，有效引入社会资本。编制重大项目“三年滚动计划”，积极争取国家专项建设基金。探索引进基金公司来钦设立私募基金。全面开展小额贷款保证保险试点，加快建立市县（区）联动融资担保体系。积极培育企业上市，推动一批企业在新三板挂牌。继续实施“引金入钦”。推进社会信用体系建设。

（三）实施创新驱动行动

加快完善技术创新服务体系。加强技术创新平台建设，以中马产业园、高新区为重点，全面实施高新技术产业培育工程，建设一批重点实验室、工程技术研究中心、企业技术中心和研发中心。培育和发展风险投资、创业投资基金，完善中小企业技术创新公共服务平台，加快科技企业孵化器布局建设。加强知识产权保护，鼓励产学研一体化发展。着力突破人才瓶颈，继续实施柔性引才“海鸥计划”，启动“助企聚才”工程，加快北部湾国际人才创业基地建设，推进博士后创新实践基地建设，创建高校毕业生创业孵化基地。推动大众创业万众创新，完善创业支持政策体系和服务机制，开展众包、众扶、众筹等创新试点，加快发展“互联网+”创业网络体系，扶持市场化、专业化、网络化众创空间建设，建设一批小微企业创业创新基地，支持农民工返乡创业，引导大学毕业生创业创新。

（四）大力发展知识经济

注重知识化、科技化、信息化，进一步完善经济知识基础设施，实施“互联网+”发展战略，完成卓能新能源整体转移，争取落户互联网安全产业、北斗卫星产业应用、3d打印、光电产业园等项目，重点发展新一代信息技术、生物技术、新医药、新能源、新材料、节能环保、智能制造等知识型产业。争取创建国家级高新区，加快推进高新区二期建设，重点打造电子、光电、医药、汽车、机电、服务产业园等“园中园”，扶持现有企业稳产达产。规划建设新型产业园，启动机器人制造产业园、深圳产业园、新能源汽车产业园等开发建设，争取机器人制造、石墨烯等项目布局，促进新兴产业集聚。新增高新技术企业5家。

六、坚持绿色发展，改善生态环境

（一）实施环境提升行动

严格减排治污，加快推进污水管网建设，新开工建设河东河西污水处理厂联动工程、垃圾处理场综合整治工程，推进河东、大榄坪污水处理厂配套管网工程，加大生活污水直排口截污建设，全面整治“黑臭水体”。完善“两高”行业调整退出目录和鼓励政策，实行电力、钢铁、造纸等行业主要污染物排放总量控制。加强减排监测体系能力建设，推进机动车污染减排、规模化畜禽养殖减排。继续实施“绿满八桂”造林绿化工程，综合治理水土流失，森林覆盖率保持在53%。申报建设滨海新城红树林湿地公园。开展水污染、大气污染、土壤污染和近岸海域污染防治，加强茅尾海、沿海区域及钦江、大风江、茅岭江等入海河流流域整治，加强pm10、pm2.5数据监测及预警，持续改善空气质量。深入开展生态文明示范区创建，优化生态空间格局，强化资源开发生态监管，保护好特殊海洋生态景观、海洋生态系统。持续推进“美丽钦州”乡村建设，深入推进生态乡村建设活动，集中精力抓好“村屯绿化”“饮水净化”“道路硬化”“公路果化”四个专项活动。

（二）大力发展生态经济

以生态化改造传统产业，培育发展新兴生态产业，大力发展特色生态产业。推动铁合金、造纸及林木加工等传统产业生态化改造，加快石化产业园等园区循环化改造，建设中马产业园国家级低碳产业园区试点，启动建设三墩循环经济示范岛，加快推动企业节能技术改造，推广太阳能光电建筑一体化技术，培育发展一批循环经济示范企业。探索建立循环经济发展基金，全面促进资源节约集约利用，加强工业中水回用，推进微型喷灌等高效节水灌溉技术，大力发展生态农林业、生态养殖业，继续推进森林生态效益补偿，打造富硒农产品基地。

七、坚持民生优先，促进和谐共享

（一）实施精准脱贫行动

全面落实“六个精准”要求，推进基础设施建设、特色产业富民、教育培训就业、金融创新和产权收益扶贫等脱贫攻坚行动。进一步改善贫困地区生产生活条件，继续创新扶贫培训工作，增强贫困村农民就业技能，完善财政专项扶贫资金分配使用监管机制，创新金融扶贫产品，整合扶贫资源，动员全社会力量更多参与扶贫工作。完成“十二五”119个贫困村的考评验收。确保年内实现8.25万人脱贫、120个贫困村摘帽。

（二）注重保障改善民生

继续实施为民办十件实事工程。加快北部湾大学筹备工作，启动二期建设。推进学前教育新一期三年行动计划，进一步优化义务教育学校布局，加快解决“入园难”和义务教育“大通铺”“大班额”问题，促进县域义务教育均衡发展。推进普通高中优质特色发展，加快现代职业教育体系建设。大力发展民办教育，推进市外国语学校迁建等项目。持续深入开展全国文明城市、国家卫生城市、广西食品安全城市创建活动，推进列入“海上丝绸之路”世界文化遗产城市名单。全面推进博物馆、图书馆、群艺馆等公共场馆建设。加大实施文化惠民工程，广泛开展群众性文化体育活动。提升卫生和计划生育服务水平，落实新的计划生育政策，完善城乡居民大病保险，加大城乡医疗救助。提高社会保险保障水平，推进精准救助，加强农村五保供养机构建设，探索农村五保供养机构公建民营模式。强化就业创业服务，落实创业扶持政策，加大职业技能培训，城镇登记失业率控制在4.5%以内。实施农村饮水安全提升工程、中小水利设施修复工程，新增恢复改善灌溉面积3万亩，50户以上自然村道路通畅率60%以上。继续做好保障性住房及农村危房改造工作。

（三）提升安全发展水平

落实安全生产监管责任，强化食品药品安全监管，推进水上搜救管理体系建设，提高防震减灾能力。健全社会矛盾纠纷调解机制，依法解决群众的合理诉求。推进司法队伍正规化、专业化、职业化建设。加强社会治安防控体系建设，强化网格化管理，推进社会治安防控工作的科技化、信息化。加强人员和装备保障，提高预防和打击犯罪的能力和水平，解决好群众反映强烈的治安问题，努力提高公共安全维护能力，深入推进平安钦州建设。

八、坚持法治思维，加强政府自身建设

实施法治建设行动，深入推进依法行政，全面履行政府职能，规范行政权力，强化严格执法，加快建设法治政府，推进治理体系和治理能力现代化，尽快提高新常态下推动发展的能力和水平。进一步规范完善重大行政决策程序，完善重大行政决策风险评估机制，充分发挥咨询专家、政府法律顾问作用。建立健全重大行政决策后评估机制和责任追究机制。全面推进政务公开，加强电子政务平台建设。健全完善行政权力方面的规章制度，自觉接受人大监督、政协民主监督，支持司法机关的依法监督，强化行政机关内部监督，加强行政监察和审计等专门监督，推进舆论监督和社会监督。巩固“三严三实”专题教育的成果，进一步改进作风。严格落实党风廉政建设主体责任，履行“一岗双责”，坚定不移推进政府系统党风廉政建设和反腐败工作。

各位代表！实现“十三五”良好开局需要各方面共同努力，要继续发展老龄事业，依法保障妇女儿童和残疾人的合法权益，支持工会、共青团、妇联等群团组织开展工作，做好国防动员、民兵预备役、双拥优抚和海防、人防、打私等工作，大力促进民族团结，依法管理宗教事务。

各位代表！“十三五”和今年经济社会发展任务十分繁重，责任十分重大。让我们紧密团结在以习近平同志为总书记的党中央周围，开拓创新，奋发有为，为实现“两个建成”宏伟目标而努力奋斗！
